# Epidemiology and Risk Factors of Tooth Loss among Iranian Adults: Findings from a Large Community-Based Study

**DOI:** 10.1155/2013/786462

**Published:** 2013-10-28

**Authors:** Saber Khazaei, A. H. Keshteli, Awat Feizi, Omid Savabi, Peyman Adibi

**Affiliations:** ^1^Dental Students' Research Center, Isfahan University of Medical Sciences, Isfahan 81749-73461, Iran; ^2^Department of Medicine, University of Alberta, Edmonton, AB, Canada T6G 2E1; ^3^Integrative Functional Gastroenterology Research Center, Isfahan University of Medical Sciences, Isfahan 81749-73461, Iran; ^4^Department of Biostatistics and Epidemiology, School of Public Health, Isfahan University of Medical Sciences, Hezar Jarib Street, Isfahan 81749-73461, Iran; ^5^Department of Prosthodontics, Torabinejad Dental Research Center, School of Dentistry, Isfahan University of Medical Sciences, Isfahan 81749-73461, Iran

## Abstract

*Objectives*. To investigate the prevalence of tooth loss and different prosthetic rehabilitations among Iranian adults, as well as the potential determinants of tooth loss. *Methods*. In a cross-sectional community-based study conducted among 8094 Iranian adults living in Isfahan province, a self-administered questionnaire was used to assess epidemiologic features of tooth loss. *Results*. Thirty-two percent of subjects had all their teeth, 58.6% had lost less than 6, and 7.2% of participants had lost more than 6 teeth. One hundred and sixty-nine individuals (2.2%) were edentulous. Among participants, 2.3% had single jaw removable partial denture, 3.6% had complete removable denture in both jaws, and 4.6% had fixed prosthesis. Others reported no prosthetic rehabilitation (89.5%). In the age subgroup analysis (≤35 and >35 years old) tooth loss was more prevalent among men than women (OR = 2.8 and 1.9, resp., *P* < 0.01). Also, in both age groups, current and former smokers had higher levels of tooth loss than nonsmokers (*P* < 0.001 and *P* < 0.05, resp.). In addition, tooth loss was positively related to metabolic abnormality for age group >35 years (adjusted OR = 1.29, *P* < 0.01). *Conclusions*. Tooth loss is highly prevalent in Iranian adult population. Community programs promoting oral health for prevention of tooth loss should be considered taking into account its major determinants including lower educational level, male gender, smoking, and metabolic abnormality.

## 1. Introduction

Tooth loss is known to have an essential role in the loss of mastication and esthetics [1]. Worldwide, the prevalence of tooth loss and edentulism is high and depends on many factors [2–4]. Prevalence of tooth loss and edentulism is high in Iran [5]. The prevalence of edentulism was reported to be about 3% among 35–44-year-old Iranians, while 22% of subjects had less than 20 teeth [6]. 

Food choice, diet, and nutrition intake can be influenced by the number and condition of teeth [1]. Inadequate dentition can cause problems in food intake; it will affect mastication, and masticatory abilities have been known to play an important role in digestive system and overall health condition [1]. Complete edentulous people were found to be at a higher risk of poor nutrition and weak chewing ability [7].

According to the World Health Organization (WHO), adults should have a minimum of 21 functional teeth to provide the ability to experience a good dietary intake without the need for dentures [8]. It has been shown that edentulism considerably reduces the quality of life [9]. Slade and Spencer [10] reported that compared to dentate people, edentulous ones experienced more social and psychological impacts on their quality of life including feeling self-conscious and avoiding social interactions. Also, edentulous subjects reported to experience more pain and discomfort and had frequent difficulties with chewing and eating [10]. 

Social-behavioral risk indicators may play a substantial role in edentulism. Potential risk factors for edentulism are low level of education, older age, gender, and marital status [11]. Burt et al. [12] evaluated risk factors of tooth loss over a period of 28 years and found that the effect of social-behavioral risk factors was more evident in the complete edentulous individuals compared to the group with partial edentulism. Low income has also been suggested to be a risk factor for edentulism [13, 14]. Caries experience, attachment loss, and cigarette smoking are other major risk indicators of tooth loss [15, 16]. In addition, patterns of tooth loss vary by gender and population [11, 16].

The major purpose of dental restorations is to replace missing teeth. A restoration is the general term for any material or prosthesis that replaces the lost tooth structure, teeth, or oral tissues [3]. Dental restorations are fixed or removable and are termed fixed restorations and removable dental prosthesis. The removable dental prostheses are also classified into removable partial denture and complete denture [3]. Based on previous studies, social and geographical variations in prosthetic replacement may be related to differences in both patients' and dentists' attitudes towards oral health as well as socioeconomic status [13, 17–19].

To our knowledge, data on the prevalence of tooth loss and its risk factors and prosthetic rehabilitation according to sociodemographic information and the prevalence of each type are sparse, particularly in Iran [6]. Previous studies among Iranian population showed a high rate of tooth loss and edentulism [3, 5, 6], and tooth loss-related risk factors have not been evaluated in a community-based survey. Therefore, the aim of the present study was to investigate the epidemiology of tooth loss and its related social-behavioral factors and to assess the prevalence of different prosthetic rehabilitation among a large sample of Iranian adults.

## 2. Materials and Methods

### 2.1. Study Design and Population

This project is a part of the “Study on the Epidemiology of Psychological, Alimentary Health and Nutrition” (SEPAHAN) [20]. In this cross-sectional study, staffs of Isfahan University of Medical Sciences (IUMS) working in university campus, hospitals, and health centers across the Isfahan province were included. In the first phase of SEPAHAN, 8094 participants completed self-administered questionnaires about demographic information, medical history, anthropometric measures, lifestyle, and nutritional factors, as well as dental status (average response rate for all questionnaires: 86.16% and response rate for dental questions: 97.5%). Detailed information about the project has been provided elsewhere [20].

### 2.2. Ethical Approval

This study approved by the Regional Bioethics Committee of Isfahan Province (numbers 189069, 189082, and 189086) and was conducted in accordance with the national and international codes.

### 2.3. Assessment of Studied Variables

Questions were selected from standard questionnaires that were previously validated in Iranian settings. However, we had to design some new assessment tools or translate some questions into Persian using the forward and back-translation procedure. The face and content validity of the final questionnaire was evaluated [20].

### 2.4. Main Variables (Tooth Loss and Restorative Replacement)

There were some questions to assess tooth loss, presence of edentulism, and the type of restorative replacement, if present. Edentulism was defined as subjects with “no remaining teeth.” Partial tooth loss was categorized into two subcategories: (1) less than 6 lost teeth and (2) 6 or more lost teeth. The following four categories were considered regarding the restorative replacement: (1) without any prosthesis, (2) single jaw removable partial dentures, (3) complete removable denture in both jaws, and (4) fixed prosthesis.

### 2.5. Other Variables

The information about age, gender, weight, and height was provided by self-administered questionnaires. Body mass index (BMI) was calculated by dividing weight in kilograms by height in meters squared. Based on self-reported data on smoking habits subjects were categorized as nonsmokers, former smokers, or current smokers. Also they were categorized as diploma (12 years formal education) and lower, bachelor, master, or doctorate based on educational attainments. Self-reported history of any predisposing chronic diseases including diabetes mellitus, hypertension, and hyperlipidemia was explored. A binary variable referred to as “metabolic abnormality” was considered to be present when a subject's BMI was ≥30 or he/she reported to have at least one of the followings: diabetes mellitus, hypertension, or hyperlipidemia. 

### 2.6. Statistical Analysis

Quantitative and qualitative variables were represented as mean ± SD and number (percent), respectively. To investigate the relationship between qualitative risk factors and levels of tooth loss, chi-square test were used as a univariate method. Multinomial logistic regressions was used as the multivariable method to investigate the association between levels of tooth loss (as dependent variable), age, educational attainment, sex, smoking habits, and metabolic abnormality as the potential determinants. Also, age stratified analysis (≤35 and >35 years old) was done using binary logistic regression for evaluating the association between tooth loss and educational attainment, sex, smoking habits, and metabolic abnormality. To compare the quantitative variables across the levels of tooth loss, one-way analysis of variance (one-way ANOVA) was conducted. All statistical analyses were performed using SPSS version 16 (SPSS Corp., Chicago, IL, USA), and *P* value less than 0.05 was considered statistically significant. 

## 3. Results

Out of 7893 respondents, 3338 (42.3%) were male and 4555 (57.7%) were female. The mean age was 37.02 ± 8.1 years with range of 19 to 75 years. Overall, 2512 (32%) of subjects had all their teeth, 4603 (58.6%) had less than 6 lost teeth, and 556 (7.2%) had 6 or more lost teeth. One hundred and sixty-nine (2.2%) individuals were edentulous. Among 7654 studied participants who responded to the prosthetic rehabilitation question, 2.3% had single jaw removable partial denture, 3.6% had complete removable denture in both jaws, and 4.6% had fixed prosthesis. Others reported no prosthetic rehabilitation (89.5%). Among the edentulous subjects, 2.5% had single jaw removable partial denture, 87% had complete removable denture in both jaws, 6.2% had implant supported fixed prosthesis, and only 4.3% of them were without dentures.  Table 1 shows the distribution of number of missing teeth across different age groups in the study population. Subjects in the upper categories of age were more likely to be edentulous or have more number of missing teeth (*P* < 0.0001). 

Tooth loss and edentulism were significantly more common in males than in females (*P* < 0.0001). Current smokers and those suffering from chronic diseases such as diabetes, hypertension, or hyperlipidemia were more likely to have higher number of tooth loss (*P* < 0.0001). In addition, less educated people were more likely for being in higher levels of tooth loss. There were significant differences between tooth loss groups in terms of age and BMI, in which those with higher levels of tooth loss had significantly higher BMI and were older (*P* < 0.0001) (Table 2).

Tooth loss and edentulism were more prevalent among current and former smokers (*P* < 0.0001). Logistic regression analysis showed that the nonsmokers were more likely to have all their teeth (adjusted OR = 12.8, 95% CI: 6.78–24.16, *P* < 0.0001), while men and less educated participants were less likely to have all teeth or lower levels of tooth loss. Age was negatively associated with lower levels of tooth loss (Table 3).

The results of multivariable logistic regression stratified based on two age groups (≤35 and >35 years old) are presented in Table 4. In both age groups, smoking and male gender were associated with increased risk of tooth loss. Also, in subjects who were above 35 years old, participants with metabolic abnormality were at 29% increased risk of losing 6 or more teeth (adjusted OR: 1.29, 95% CI: 1.07–1.59, *P* < 0.01) (Table 4).

## 4. Discussion

In this large cross-sectional community-based study in Iran, the prevalence of tooth loss was found to be high among the study population. Only one-third of the population did not experience tooth loss, whereas about one-tenth of participants showed significant loss (over 6 lost teeth). Also, the prevalence of edentulism was found to be 2.2%. 

Various prevalence rates of tooth loss have been reported around the world. Based on Swiss Health Survey among 14326 subjects, the prevalence of edentulism was 0.3% and 26.8% in 15–24-and 65–74-year-old subjects, respectively [21]. According to a systematic review conducted in Iran, the prevalence rate of tooth loss varied from 0.3% in 3–5 year-old children to 70.7% in adults older than 65 [5]. In a large population-based study among 35–44-year-old Iranians, the prevalence of edentulism was estimated to be 3% [6]. In a study carried out among 1545 Turkish elderly, the prevalence of edentulism was 48% [22]. A systematic review of 73 studies in Europe showed that, in many European countries, edentulism is already rare among people of working age or up to 60 years of age, whereas there are still many edentulous subjects in the age group above 65—in studies from the 1990s, the prevalence varied between 15% and 72% [11]. 

In contrast to several studies, the present study showed that the tooth loss and edentulism were more prevalent in males [6, 11, 23]. It has been discussed that the greater importance of esthetics among women may be associated with higher rate of edentulism [5]. The high prevalence rate of tooth loss and edentulism among men seems to be dental disease-related such as dental caries, periodontal disease, trauma, infection, and sociobehavioral including smoking and poor oral hygiene. Many studies indicated that tooth loss and edentulism are positively associated with aging, which is similar to the results of the present study [5, 11, 24, 25]. Periodontitis is one of the major risk factors of tooth loss [26], and the prevalence of periodontitis is increased by aging [25]. This can be considered as logical item in aging-related tooth loss.

Previous studies have indicated that cigarette smokers are more inclined to missing teeth and edentulism [27–29]. Dental caries are more prevalent in smokers which may result in loss of teeth [30]. Ojima et al. [31] reported a significant positive exposure-related relationship between smoking and the rate of tooth loss. Axelsson et al. [27] demonstrated a significant larger number of decay and filled teeth among smokers than nonsmokers. In a cross-sectional study among 1002 Japanese young adult women, a positive association was reported between active smoking and tooth loss [32]. A study in Finnish population reported a significant relationship between smoking and loss of six or more teeth, which was similar to our study [33]. Furthermore, Dietrich et al. [34] reported that cigarette smoking was strongly associated with a dose-dependent increase in the risk for incident tooth loss among male health professionals. Saliva has an important effect on the maintenance of oral health and buffers the acids when produced. Saliva also plays an important role in self-cleansing of the teeth and physically removes debris from tooth surfaces. In addition, it has immunological and bacteriostatic properties [35, 36]. Heintze [37] demonstrated that for cigarette smokers, the buffering capacity of saliva was substantially lower than nonsmokers and that the number of *Lactobacilli* and *Streptococcus mutans* was significantly higher in saliva of smokers than nonsmokers. The decrease in the buffering capacity of saliva and increase in the level of cariogenic bacteria result in high incidence of caries in smokers, which eventually leads to tooth loss. In the present study, we demonstrated that cigarette smoking can be considered as a risk factor of tooth loss and edentulism. It should be noted that the prevalence of smoking in the study population was much lower than its prevalence in Isfahan province. The study population of the present study consisted of a medical university staff with higher educational level than the Iranian general population, and it has been shown that university educational level was shown to be inversely related to smoking in Isfahan [38]. 

In the present study, we found a positive relationship between tooth loss and BMI. It has been suggested that the number of teeth may have an effect on BMI [39]. Conflicting results have been reported regarding the relationship between number of teeth and BMI. In several studies lower intake of recommended nutrients has been reported in edentate subjects in comparison to dentate subjects [40–42]. Sheiham and Steele [1] reported that having any number of remaining teeth is better than having none when comparing the nutrient intake of individuals with very few teeth as opposed to edentulous subjects. Having at least 20 teeth or more helps the intake of nutrients, prevention of underweight and overweight conditions, and prevention of abnormal BMI [8]. Ostberg et al. [43] found statistically significant associations between a small number of teeth (less than 20) and BMI, waist-hip ratio, and waist circumference as different indicators of obesity. However, it should be noted that prosthetic replacement of lost teeth does not necessarily guarantee the prevention of undesired BMI. In a study conducted by Hilgert et al. [44] partial or complete tooth loss not being replaced with prosthetics was in relation with obesity.

In the present study, a binary variable referred to as “metabolic abnormality” was created when at least one of these four conditions was present: diabetes mellitus, hypertension, hyperlipidemia, or obesity (BMI ≥ 30). The results showed that subjects with metabolic abnormality were more prone to have higher level of tooth loss and being edentulism. Diabetes mellitus has been known as a risk factor for periodontitis [45]. Patients with diabetes show an increase in proinflammatory cytokines in the gingival fluid and tissues compared to periodontitis patients without diabetes [46]. The presence of periodontal pathogens in diabetic patients can initiate a cycle of tissue destruction and impaired wound healing which lead to tooth loss [47]. In addition, total tooth loss has been associated with increased levels of systolic blood pressure among Brazilian adults [48] and has been introduced as a risk indicator for hypertension in South Africa [49]. It has been suggested that periodontal disease is a possible risk factor for dyslipidemia [50, 51]. The four elements of “metabolic abnormality” in the present study (obesity, diabetes mellitus, hypertension, and dyslipidemia) can be attributed to the major determinants of “metabolic syndrome.” Therefore, it is highly suggested to perform further studies to investigate the relationship between metabolic syndrome and tooth loss and the possible mechanisms. 

In the present study, the prevalence of tooth loss and its related risk factors was evaluated using a self-administrated questionnaire, which subsequently can affect the results due to probable misclassifications. Lack of clinical evaluations is another weakness of our study. Tooth loss and edentulism are highly prevalent among elderly. Since we have recruited a group of apparently healthy adults with the mean age of almost 37 years, the prevalence of these conditions in Iranian adult population may have been underestimated in the present study.

## 5. Conclusion

In the present study, we showed a high prevalence of tooth loss among Iranian adults. Aging, male gender, low level of education, cigarette smoking, and presence of metabolic abnormality can be considered as risk factors of tooth loss and edentulism. This study highlighted that among the studied potential risk factors cigarette smoking had the major impact on tooth loss. Further prospective studies are required to evaluate the possible association between metabolic syndrome and tooth loss. Community programs directed toward promoting oral health and multidisciplinary efforts are suggested for prevention of tooth loss. More longitudinal studies are needed to determine the main risk factors of tooth loss and its possible consequences among Iranian adults.

## Figures and Tables

**Table 1:** Distribution of the number of missing teeth across the different age groups.

Age group	Number (%) of lost teeth			
	0	<6	≥6	Edentulous
19–24	182 (61.3)	113 (38.8)	2 (7)	0 (0)
25–29	639 (54.2)	538 (45.6)	2 (2)	0 (0)
30–34	608 (39.9)	874 (57.4)	38 (2.5)	3 (2)
35–39	412 (27.7)	986 (66.1)	78 (5.2)	15 (1)
40–44	294 (21.7)	888 (65.5)	139 (10.3)	35 (2.6)
45–49	125 (15.9)	497 (63.3)	121 (15.4)	42 (5.4)
50+	80 (13.1)	336 (55.3)	122 (20.1)	70 (11.5)

**Table 2:** Distribution of the number of missing teeth across different categories of demographic and clinical characteristics.

Characteristics	Number of lost teeth				
	0	<6	≥6	Edentulism	
Sex					
Male	726 (22.6%)	1997 (61.5%)	370 (11.5%)	142 (4.4%)	*P* < 0.001^a^
Female	1712 (38.5%)	2522 (56.7%)	187 (4.2%)	25 (0.6%)	
Age	33.3 ± 7.3	37.7 ± 7.7	44.0 ± 6.8	47.9 ± 6.9	*P* < 0.001^b^
Education					
Diploma and lower	640 (19.8%)	2029 (63%)	414 (12.9%)	137 (4.3%)	*P* < 0.001^a^
Bachelor	1556 (40.3%)	2169 (56.1%)	118 (3.1%)	21 (0.5%)	
Master and doctorate	274 (45.4%)	316 (52.2%)	13 (2.1%)	2 (0.3%)	
Body mass index	24.4 ± 7.7	25.3 ± 4.1	25.8 ± 4.3	25.8 ± 4.7	*P* < 0.001^b^
Smoking					
Former smokers	38 (10.7%)	209 (58.9%)	81 (22.8%)	27 (7.6%)	*P* < 0.001^a^
Nonsmokers	2154 (34.5%)	3697 (59.2%)	327 (5.2%)	68 (1.1%)	
Smoker**s**	27 (8.7%)	148 (47.9%)	75 (24.3%)	59 (19.1%)	
Hyperlipidemia					
Yes	134 (20.8%)	414 (64.2%)	76 (11.8%)	21 (3.3%)	*P* < 0.001^a^
No	2378 (33%)	4189 (58.1%)	490 (6.8%)	148 (2.1%)	
Hypertension					
Yes	56 (19.2%)	190 (65.1%)	35 (12%)	11 (3.8%)	*P* < 0.001^a^
No	2456 (32.5%)	4413 (58.4%)	531 (7%)	158 (2.1%)	
Diabetes mellitus					
Yes	27 (16.3%)	104 (62.7%)	28 (16.9%)	7 (4.2%)	*P* < 0.001^a^
No	2485 (32.3%)	4499 (58.6%)	538 (7%)	162 (2.1%)	

^a^Chi-square test.

^b^One-way ANOVA.

**Table 3:** Crude and adjusted odds ratios and 95% confidence intervals for the independent potential risk factors of tooth loss based on multinomial logistic regression analysis.

	Tooth loss status*											
	0				<6				≥6			
	Crude OR (95% CI)	*P* value	Adjusted OR (95% CI)	*P* value	Crude OR (95% CI)	*P* value	Adjusted OR (95% CI)	*P* value	Crude OR (95% CI)	*P* value	Adjusted OR (95% CI)	*P* value
*Age *	0.79 (0.76–0.82)	<0.001	0.803 (0.781–0.827)	<0.0001	0.85 (0.82–0.88)	<0.001	0.865 (0.841–0.889)	<0.0001	0.94 (0.91–0.97)	<0.0001	0.944 (0.918–0.972)	<0.0001

*Smoking *												
Former Nonsmoker Smoker (ref)	2.68 (1.41–5.06) 6.7 (4.1–10.9)	0.002 <0.0001	2.19 (1.01–4.7) 12.8 (6.78–24.16)	0.046 <0.0001	2.91 (1.82–4.66) 21.7 (15.02–31.35)	<0.0001 <0.0001	2.48 (1.41–4.36) 9.14 (5.6–14.92)	<0.0001 <0.0001	2.03 (1.21–3.42) 3.6 (2.4–5.41)	0.007 <0.0001	2.273 (1.25–4.11) 3.35 (1.98–5.68)	0.0007 <0.0001

*Education *												
Diploma and lower Bachelor Master and doctorate (ref)	0.03 (0.008–0.129) 0.54 (0.12–2.32)	<0.0001 0.409	0.024 (0.006–0.102) 0.195 (0.043–0.891)	<0.0001 0.035	0.083 (0.021–0.338) 0.65 (0.151–2.78)	0.001 0.560	0.087 (0.02–0.365) 0.370 (0.082–1.678)	<0.0001 0.197	0.43 (0.091–1.958) 0.89 (0.188–4.28)	0.279 0.888	0.509 (0.108–2.399) 0.795 (0.156–4.03)	0.393 0.782

*Sex *												
Male Female (ref)	0.07 (0.048–0.609)	<0.0001	0.439 (0.252–0.764)	0.004	0.13 (0.088–0.199)	<0.0001	0.597 (0.347–1.029)	0.063	0.34 (0.218–0.529)	<0.0001	0.891 (0.498–1.594)	0.697

*Metabolic abnormality (ref: yes) *	3.38 (2.41–4.73)	<0.0001	1.18 (0.77–1.82)	0.45	1.93 (1.40–2.67)	<0.0001	1.17 (0.78–1.75)	0.46	1.18 (0.82–1.70)	0.37	1.07 (0.69–1.65)	0.77

*Dependent variable in multinomial logistic regression with four categories; edentulism was considered as reference category.

Ref: reference category; OR: odd ratio; 95% CI: 95% confidence interval.

**Table 4:** Age stratified analysis of association between tooth loss^1^ and metabolic abnormality, smoking, and gender.

Independent variables	Age ≤ 35 (*n* = 3086)	*P* value	Age > 35 (*n* = 4807)	*P* value
	OR (95% CI)^2^		OR (95% CI)	
Metabolic abnormality^3^				
Yes	1.57 (0.67–3.68)	0.29	1.29 (1.07–1.59)	0.009
No (ref)^4^	1.00		1.00	
Smoking habit				
Former smoking	3.43 (1.85–5.0)	0.02	1.49 (1.05–2.05)	0.02
Current smoking	4 (1.28–6.74)	<0.0001	5.29 (4.00–6.66)	<0.0001
Nonsmoking (ref)	1.00		1.00	
Sex				
Male	2.8 (1.43–4.16)	0.002	1.9 (1.5–2.33)	<0.0001
Female (ref)	1.00		1.00	

^1^A binary variable was created for tooth loss: (i) 0–5 lost teeth and (ii) ≥6 lost teeth.

^2^OR: odd ratio and 95% CI: 95% confidence interval have been obtained from binary logistic regression; the category of 0–5 lost teeth was considered as the reference category.

^3^“Metabolic abnormality” was considered to be present when a subject's body mass index was ≥30 or he/she reported to have at least one of the following: diabetes mellitus, hypertension, or hyperlipidemia.

^4^Ref: reference category.
